# *Penicillium aculeatum* pre-activated with cellulose and different phosphorus sources promotes maize growth, nutrition and mycorrhiza formation, and decreases *Microdochium bolleyi* root infection

**DOI:** 10.1007/s00284-026-05098-4

**Published:** 2026-08-26

**Authors:** John Larsen, Aikaterini Efthymiou, Mette Haubjerg Nicolaisen, Birgit Jensen, Ole Nybroe, Beatriz Gómez-Muñoz

**Affiliations:** 1 Section for Microbial Ecology and Biotechnology, Department of Plant and Environmental Sciences, University of Copenhagen, Thorvaldsensvej 40, Frederiksberg, 1871 Denmark; 2 Instituto de Investigaciones en Ecosistemas y Sustentabilidad, Universidad Nacional Autónoma de México, Antigua Carretera a Pátzcuaro 8701, Morelia, 58190 México; 3 Section for Plant and Soil Science, Department of Plant and Environmental Sciences, University of Copenhagen, Thorvaldsensvej 40, Frederiksberg, 1871 Denmark; 4 Present Address: Microbe and Culture Research, R&D, Novonesis, Hørsholm, Denmark; 5 Present Address: Department of Biotechnology and Environmental Protection, Estación Experimental del Zaidín, Spanish Council for Scientific Research (CSIC), Profesor Albareda 1, Granada, 18008 Spain

## Abstract

Microbial plant growth promoters often perform inconsistently under natural soil conditions most likely due to their poor establishment after inoculation. Here, we conducted a growth chamber pot experiment with maize using pre-activated inoculum of the P-solubilizing fungus *Penicillium aculeatum* to facilitate its establishment in a natural non-sterile agricultural soil. Hence, *P. aculeatum* was preincubated for one week in soil amended with cellulose as a C source and with different P sources, i.e. sewage sludge ash, Ca_3_(PO_4_)_2_, and no P fertilization as control. Control treatments of C and P sources without *P. aculeatum* were also included. The pre-activated inocula were mixed into natural agricultural soil (1:40 w/w), maize seeds were sown, and plants were grown for 42 days. Measured variables included shoot and root dry weight, nutrient content, and root colonization by arbuscular mycorrhizal fungi (AMF) and root infection with *Microdochium bolleyi*. Overall, *P. aculeatum* inoculation enhanced maize growth, nutrient content and AMF root colonization, while reduced root infection with *M. bolleyi*, independent of P source used. Both P sources increased maize growth and nutrient content, though sewage sludge ash showed stronger effect than Ca_3_(PO_4_)_2_. None of the P sources affected AMF root colonization, but sewage sludge ash decreased *M. bolleyi* root infection and increased the population density of *P. aculeatum*. In conclusion, inoculation with pre-activated *P. aculeatum* (with cellulose and P sources) enhances maize growth and nutrient uptake, and is associated with increased AMF root colonization and reduced root infection by *M. bolleyi*.

**Supplementary Information:**

The online version contains supplementary material available at https://doi.org/10.1007/s00284-026-05098-4.

## Introduction

Phosphorus (P) is an essential nutrient in agriculture, and its availability limits crop yield in many agroecosystems. Plant P availability in soil depends on different processes including absorption-desorption, mobilization of Ca-, Fe-, and Al- phosphates, dissolution from rock or mineralization from organic pools [1]. However, these processes do not fully satisfy the P requirement of crops and P fertilizers need to be applied to ensure crop productivity [2]. Mineral P fertilizers are produced from rock phosphate, which is a non-renewable resource with the majority of the reserves located in Morocco, China, and the United States [3, 4]. Alternative renewable P sources include compost, manure, biosolids and sewage sludge ash [5].

The use of P solubilizing microbial plant growth promoters, which may be native soil populations and/or commercial inoculants, represent a complementary strategy for improving P availability in agricultural soil [6, 7]. Many soil microorganisms including bacteria and fungi can solubilize P from otherwise poorly available forms [7, 8]. Among these, *Penicillium* spp. are common saprotrophic soil fungi [9], which are key players in soil biogeochemical P cycling due to their P solubilizing traits involving the production of organic acids [10]. Commercial *Penicillium* inoculants are available as plant growth promoting biofertilizers in agriculture to improve crop P use efficacy [11, 12]. The commercial biofertilizer Jumpstart is based on *Penicillium bilaiae*, which is used as a seed coat in cereals such as wheat and maize [13]. Previous studies have shown that *Penicillium aculeatum* can solubilize P from Ca_3_(PO_4_)_2_ or sewage sludge ash in natural agricultural soil [14], and can improve the P fertilizer value of biochar for wheat grown in gamma-irradiated semi-sterile soil [15].

A major limitation of microbial inoculants, however, is their inconsistent establishment and performance in natural soils. Carriers containing C sources such as simple sugars, starch, skimmed milk powder and cellulose have been used to promote the establishment of commercial microbial inoculants, including P solubilizing fungi in different agricultural environments [7, 14, 16–18]. Recent work showed that the addition of cellulose enhanced the establishment and persistence of *P. aculeatum* in natural agricultural soil and increased P solubilization from sewage sludge ash in a soil incubation experiment [14]. However, whether this increased P solubilization translates into improved plant P nutrition and growth yet needs to be examined.

When introducing plant beneficial microbial inoculants such as *Penicillium* spp. to agroecosystems it is important to consider their possible effects on other root and soil microbiota. Arbuscular mycorrhizal fungi (AMF), which are common root inhabitants of most crops, are known to facilitate plant nutrient acquisition especially P and other immobile plant nutrients [19] and are also known to reduce soil N and P losses [20]. Furthermore, AMF are known to promote plant health via induction of disease and pest tolerance, as well as direct competition with root-inhabiting pathogens [21, 22]. On the other hand, it is also important to consider the possible effects of *Penicillium* spp. on other root-inhabiting microorganisms such as *M. bolleyi*, which is a common root-inhabiting fungus in cereals like maize [23]. In a study with wheat using gamma irradiated soil dual inoculation with *P. aculeatum* and the AMF *Rhizophagus irregularis* resulted in increased root colonization by the AMF and increased population density of *P. aculeatum* [24], but the effects of *P. aculeatum* on native populations of AMF and other root-inhabiting fungi such as *M. bolleyi* in natural agricultural soil remain to be examined.

The objective of this study was to investigate the effects of applying inoculum of the P solubilizing fungus *P. aculeatum* that was pre-activated with cellulose as a C source and sewage sludge ash and Ca_3_(PO_4_)_2_ as P sources on maize plant growth and nutrient content and, on root colonization with mycorrhizal fungi and other root-inhabiting fungi such as *M. bolleyi* under controlled growth chamber conditions in a natural (non-sterile) agricultural soil. We hypothesize that (1) inoculation with *P. aculeatum* pre-activated with cellulose as a C source would promote maize plant growth and P nutrition due to the solubilization of P from different P sources (Ca_3_(PO_4_)_2_ and sewage sludge ash), and (2) successful establishment of *P. aculeatum* in soil would affect root colonization of native root associated fungi such as AMF and *M. bolleyi.*

## Materials and methods

### Soil

A sandy loam soil containing 16.4% clay, 17.3% silt, 33.3% fine sand and 31.2% coarse sand classified as luvisol (FAO, 2015), was collected from the Long-term Nutrient Depletion Trial (LTNDT) on the experimental research farm of the University of Copenhagen in Taastrup, Denmark (55°40′N, 12°17′E). The soil was collected from the N½K½ treatment, which had been depleted for P for 27 years and more information about the LTNDT experiment can be found in [25]. Soil pH (CaCl_2_) was 7.27, Olsen P was 5.5 mg kg soil^− 1^, water-extractable P (WEP) was 2.96 mg P kg soil^− 1^, and exchangeable K and Mg were 63.3 and 63.5 mg kg soil^− 1^, respectively. The total amount of soil C, N, P and K was 11.5, 1.55, 0.34 and 15.6 g kg soil^− 1^, respectively [25].

### Preparation of fungal spore suspension

*P. aculeatum* was obtained from the American Type Culture Collection (ATCC 10409). Cultures established from frozen spore stocks (− 80 °C) were grown on potato dextrose agar (PDA, Sigma Aldrich Co.) plates for two weeks. The spores were harvested with sterile demineralized water (Mili-Q; Millipore), and then sub-cultured for two weeks on a new PDA plate. The second-generation spores were collected by washing the plate with sterile demineralized water. The spore suspension was then filtered through sterile glass wool (Miracloth, EMD Millipore Corporation, Billerica, MA 01821 USA) and centrifuged at 4000 rpm (Kubota 5500, Kubota Corporation, Japan) and 4 °C for 10 min. The spore concentration in the suspension was determined using a hemacytometer (Improved Neubauer, Brand, Germany) and adjusted to 10^7^ conidia ml^−1^ with sterile MilliQ.

### Preparation of pre-activated *P. aculeatum* inoculum

Six different pre-activated inoculum types were prepared in individual portions of 50 g (dry weight) of sieved (< 4 mm) natural sandy soil. According to our previous study, each portion received C, N and P [14]. The C source, cellulose (microcrystalline, powder, 20 μm, CAS: 9004-34-6, 159 Sigma Aldrich) was added to the pots to reach a final concentration of 0.25% C (dry weight), and thoroughly mixed into the soil. Then, nitrogen equivalent to 60 mg N kg soil^− 1^ was added as NH_4_NO_3_ to the soil. Phosphorus sources of different availability were added at three levels: Ca_3_(PO_4_)_2_ and sewage sludge ash were applied to a final concentration of 30 mg P kg soil^− 1^, and non-P (No P) control was included. Subsequently, the mixtures were thoroughly mixed. Then, one set of pots was inoculated with 1 ml of *P. aculeatum* spore solution (+ Pa) containing 10^7^ conidia ml^− 1^ (2 × 10^5^ conidia g^− 1^ soil), whereas 1 ml of sterile MiliQ water was added to the other set of samples (-Pa). Finally, the water content in the pots was increased by adding sterile MiliQ water to reach 60% of field capacity (18.6% water w/w) (estimated using a sandbox soil water equilibration system). Four samples of each treatment were prepared, and all samples were incubated in a growth chamber at 15˚C for 7 days when the fungi were visible to the eye.

### Plant experiment in pots

For each pot, 950 g of soil (dry weight) was mixed with 1000 g of quartz sand in a plastic bag. A nutrient solution was added to the surface of the soil: sand mixture to reach the following final concentrations: 112 mg K kg soil^− 1^, 30 mg Ca kg soil^− 1^, 0.15 mg Cu kg soil^− 1^, 15 mg Mg kg soil^− 1^, 0.30 mg Zn kg soil^− 1^, 0.014 mg Mo kg soil^− 1^, 0.30 mg Fe kg soil^− 1^, 0.22 mg B kg soil^− 1^ and 0.45 mg Mn kg soil^− 1^. Then, the mixture was left to dry for two days. Subsequently, the soil: sand mixtures were thoroughly mixed again to homogenize nutrients. Later, the pre-activated soil inoculum (prepared as described before) of *P. aculeatum* (all of them amended with N and C as stated above, and treated with three P sources: No P, Ca_3_(PO_4_)_2_ or sewage sludge ash and inoculated with *P. aculeatum* (+ Pa) or non-inoculated (-Pa) were added and distributed homogeneously to the soil mixtures (1:40 w/w). Thereafter, the mixtures were transferred to 2 L pots, and the moisture content was adjusted to 60% of the field capacity (corresponding to 18.6% water w/w). Finally, two maize seeds (*Zea mays*, variety Ambition from Limagrain A/S, Horsens, Denmark), washed with distilled water, were sown in the centre of each pot at 2 cm depth, and after germination thinned to one seedling. The experiments were carried out under controlled conditions in a growth chamber with day/night of 16/8 hrs and temperature level of 20 °C, humidity levels of 60% and a light level of 280 µmol m^− 2^ s^− 1^. Water content was adjusted every two days to reach 60% of the field capacity during the first week of growing, and then increased to and maintained at 70% for the rest of the experiment.

### Shoot and root analysis

Shoot length was measured using a ruler at 14, 22, 29 and 42 days after sowing (DAS). At harvest, shoots were cut and dried at 70 °C to determine dry matter biomass, whereas the soil was washed off the root. A root subsample of 1 g was separated to determine the percentage of AMF root colonization in terms of the presence of AMF structures like hyphae, vesicles and arbuscules, and the percentage of root colonization with *M. bolleyi* in terms of the presence of dark pigmented chlamydospores [23]. Root length was determined using the grid-line-intersect method [26], whereas the rest of the root samples were dried at 70 °C to quantify root biomass. Measurement of percent root colonization by AMF was done using the line-intercept method of Giovanetti and Mosse [27] after clearing and staining the roots following the methods proposed by Kormanik and McGraw [28] except that acid fuchsin was replaced by trypan blue. In addition, percent root infection by *Microdochium bolleyi* was measured following the procedure described by Aguilar et al. [23]. Before root clearing (20 min in 10% KOH at 90 °C in a water bath) and staining (3 min at 90 °C in a water bath using trypan blue at 0.05% in lactoglycerol) roots were cut in 5–10 mm segments and mixed in a plastic container with 1 L tap water to allow taking a representative 1 g subsample. Hereafter, the stained root segments in lactoglycerol were spread out evenly in the 9 cm Petri dish marked with parallel lines with 1 cm interval in a thin layer to avoid roots from moving. Fungal root colonization/infection were measured using a stereo microscope scoring presence/absence of AMF (vesicles, arbuscules and hyphae) and *M. bolleyi* (dark pigmented spore clusters) structures in 100 root intersections with the lines in the Petri dish. Root samples were scored blind and all samples were analyzed by the same person. Percent AMF root colonization was quantified by dividing the total number of root intersections with the presence of any of the AMF structures divided by 100 and multiplied with 100. The same formula was used for quantification of *M. bolleyi* root infection scoring absence/presence of *M. bolleyi* structures. 

Shoots and roots were subsequently milled and digested with HNO_3_ and H_2_O_2_ before analysis of the content of macro and micronutrient with ICP-OES Agilent 5100.

### Quantification of *P. aculeatum* by qPCR

Rhizosphere soil was sampled from each pot at harvest (42 DAS after sowing), and the *P. aculeatum* biomass was quantified by real-time PCR (qPCR). Soil samples were freeze-dried, homogenized and stored at − 20°C. DNA was extracted from 0.5 g of soil using phenol-chloroform, as described in [29]. Quantification was performed using the primer pair ITS-F2 (5′-ACGGCTTGTGTGTTGGGTGC-3′) and Universal ITS-R (5′-ACCGTGGTAAAATGTGGTGGT-3’) [24], which amplifies the fungal internal transcribed spacer (ITS1-5.8 S and ITS2) regions. Although these primers are not species-specific per se, specificity for *P. aculeatum* was ensured by the use of a controlled experimental system inoculated exclusively with *P. aculeatum*, together with melting curve analysis and verification of the expected amplicon size.

All reactions were run on an AriaMx Real-time PCR system, using a 20 µl reaction volume containing 2 µl of soil DNA extract (either undiluted or diluted 10 times), 1 µg µl^− 1^ BSA (New England Biolabs^®^ Inc., UK), 2× Master Mix (Brilliant III Ultra-Fast QPCR Master Mix SYBR^®^ Green, Agilent Technologies, Manchester, UK) and 0.5 µM of each primer. DNA extracts were used either undiluted or diluted tenfold to minimize potential PCR inhibition caused by co-extracted soil compounds; dilution was applied when preliminary tests indicated improved amplification efficiency and more consistent Ct values.

Thermal cycling condition were as follows: 3 min at 95 °C followed by three cycles (touchdown) starting at 64.5 °C and decreasing by 0.5 °C per cycle, then 37 cycles of 5 s at 95 °C, 10 s at 63 °C, and finally one cycle of 30 s at 95 °C, 30 at 63 °C and 30 s at 95 °C. Quantification was based on a standard curve generated by inoculating tenfold dilutions (six orders of magnitude, down to 50 spore-equivalents g^− 1^ soil) of *P. aculeatum* spore solution into an identical soil matrix, followed by DNA extraction and qPCR analysis as described above. The PCR assay an efficiency of 93%. Four replicate samples per treatment were run, originating from the individual incubation containers.

### Statistics

The plant experiments were set up under a completely randomized design. Differences between the treatments for all the variables studied were tested using factorial ANOVA (including interactions) and the Tukey post hoc test. Repeated measures ANOVA was used to test the treatment effect on shoot length at 14, 22, 29 and 42 DAS. Normality and homoscedasticity were checked in all cases and significance was accepted at *p* < 0.05. Principal Component Analysis (PCA) was applied for the macro and micronutrient contents in shoot and root. All statistical analyses were performed using the software ‘R’ version 3.4.1 [30].

## Results

### Plant growth

Treatment means of the factors “Inoculation” and “P source” for shoot and root length and biomass are presented in Table 1. There were no significant interactions between “Inoculum” and “P source” for any of the three plant growth variables (Table S1, supplementary material). However, inoculation with *P. aculeatum* significantly increased the shoot length of maize plants at 29 and 42 days after sowing, while no effects were observed at early stages of growing (Table 1). At harvest, total or specific root lengths were similar for inoculated and non-inoculated plants. Similarly, root biomass was not affected by *P. aculeatum* inoculation, whereas total shoot and total plant biomass at harvest was significantly higher in plants inoculated with *P. aculeatum*. The different P sources affected plant growth; in particular, the addition of sewage sludge ash increased shoot length from day 22 and 29 until the end of the experiment, compared with the Ca_3_(PO_4_)_2_ (TCP) treatments and no P fertilization control, respectively. Similarly, the total root length, shoot, root and total biomass were also significantly higher when sewage sludge ash was added. In contrast, the addition of Ca_3_(PO_4_)_2_ had no effects on plant growth.

**Table 1:** Factor treatment means ± standard error of shoot length measured at 14, 22, 29 and 42 days after sowing (DAS), total and specific root length and shoot, root, total biomass and root: shoot biomass ratio determined at harvest for plants none inoculated (-Pa) or inoculated with *P. aculeatum* (+ Pa) and amended with three different P sources (no P, tricalcium phosphate (TCP) and sewage sludge (sludge)). Different letters indicate significant treatment differences among treatments for each factor (-Pa vs. +Pa or no P vs. TCP vs. sludge)

		Shoot length (DAS)				Root length		Biomass			
		14	22	29	42	Total	Specific	Shoot	Root	Total	Root: shoot
Inoculum	-Pa	39 ± 0.6^a^	56 ± 1.0^a^	65 ± 1.8^b^	86 ± 2.1^b^	126 ± 26^a^	80 ± 4.3^a^	3.7 ± 0.6^b^	1.5 ± 0.3^a^	5.2 ± 0.9^b^	0.40 ± 0.01^a^
	+Pa	38 ± 0.4^a^	58 ± 0.6^a^	70 ± 1.3^a^	92 ± 1.3^a^	144 ± 20^a^	86 ± 4.2^a^	4.5 ± 0.6^a^	1.7 ± 0.2^a^	6.2 ± 0.8^a^	0.37 ± 0.02^a^
P source	no P	38 ± 0.7^a^	57 ± 0.9^ab^	66 ± 1.4^b^	86 ± 1.8^b^	77 ± 9^b^	81 ± 4.5^a^	2.6 ± 0.2^b^	0.9 ± 0.1^b^	3.5 ± 0.3^b^	0.37 ± 0.03^a^
	TCP	39 ± 0.7^a^	55 ± 1.3^b^	64 ± 1.7^b^	85 ± 2.2^b^	90 ± 10^b^	78 ± 4.1^a^	2.8 ± 0.2^b^	1.1 ± 0.1^b^	3.9 ± 0.3^b^	0.41 ± 0.02^a^
	sludge	39 ± 0.6^a^	59 ± 0.6^a^	73 ± 1.0^a^	96 ± 1.0^a^	237 ± 14^a^	90 ± 6.3^a^	6.9 ± 0.3^a^	2.7 ± 0.1^a^	9.6 ± 0.4^a^	0.39 ± 0.01^a^

### AMF root colonization and *M. bolleyi* root infection

**Fig. 1:**
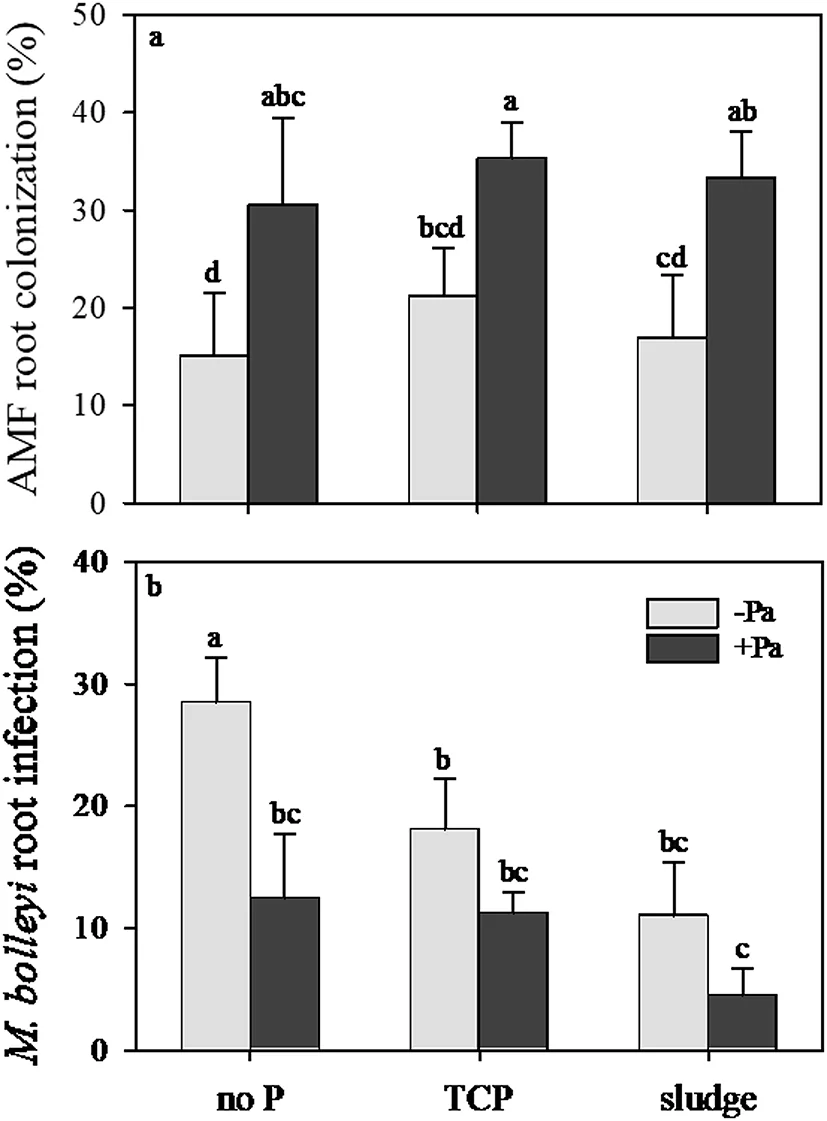
Arbuscular mycorrhizal fungi root colonization and root infection with *Microdochium bolleyi* quantified at harvest (42 DAS) for none inoculated plants (-Pa) or inoculated with *P. aculeatum* (+ Pa) and amended with three different P sources (no P, tricalcium phosphate (TCP) and sewage sludge (sludge)). Values are the mean of four replicates, and error bars denote standard error. Different letters indicate significant treatment effects for each individual interaction

Inoculation with *P. aculeatum* duplicated the percentage of root colonized by AMF compared to non-inoculated samples regardless of the addition of different P sources (Fig. 1a). AMF root colonization was similar for the three P sources used.

Plants inoculated with *P. aculeatum* showed significantly lower *M. bolleyi* root infection compared to non-inoculated plants (Fig. 1b, Table S1 supplementary material). The addition of P as Ca_3_(PO_4_)_2_ and sewage sludge ash decreased the percentage of *M. bolleyi* root infection compared to the No P treatments.

Root infection with *M. bolleyi* was negatively correlated with plant biomass as well as with macro and micronutrient contents in the shoot and root (Table 2) as well as the abundance of *P. aculeatum* measured by qPCR.

**Table 2:** Pearson-moment correlation coefficient between arbuscular mycorrhiza fungi (AMF), *Microdochium bolleyi*, and the abundance of *P. aculeatum* (Pa) measured by qPCR, and the rest of the parameters measured in this study

Variables		AMF	M. bolleyi	*P*. aculeatum abundance
Shoot height	14	0.01	−0.18	0.05
	22	0.17	−0.4	0.41*
	29	0.22	−0.65*	0.60*
	42	0.34	−0.68*	0.60*
Root length	Total	0.02	−0.59*	0.3
	Specific	0	−0.27	0.02
Biomass	Shoot	0.13	−0.69*	0.48*
	Root	0.09	−0.64*	0.42*
	Total	0.12	−0.68*	0.46*
	Root: shoot	−0.24	0.16	−0.21
Fungi	AMF	-	−0.57*	0.62*
	*M. bolleyi*	−0.57*	-	−0.64*
	*P. aculeatum abundance*	0.62*	−0.64*	-
Shoot	N	0.18	−0.72*	0.52
	P	0.11	−0.65*	0.48*
	K	0.17	−0.73*	0.51*
	Ca	0.14	−0.67*	0.45*
	Mg	0.19	−0.71*	0.56*
	S	0.23	−0.73*	0.61*
	B	0.25	−0.72*	0.62*
	Cu	0.22	−0.73*	0.58*
	Fe	0.19	−0.38	0.27
	Mn	0.02	−0.64*	0.42*
	Na	0.17	0.16	−0.13
	Zn	0.13	−0.69*	0.48*
Root	N	0.1	−0.63*	0.35
	P	−0.06	−0.48*	0.21
	K	0.09	−0.65*	0.39
	Ca	0.01	−0.55*	0.3
	Mg	0.04	−0.60*	0.36
	S	0.05	−0.61*	0.34
	B	0	−0.51*	0.29
	Cu	0.05	−0.61*	0.32
	Fe	−0.01	−0.50*	0.28
	Mn	−0.04	−0.46*	0.23
	Na	0.09	−0.62*	0.45*
	Zn	−0.07	−0.51*	0.2

* p < 0.05

### Abundance of *P. aculeatum*

**Fig. 2:**
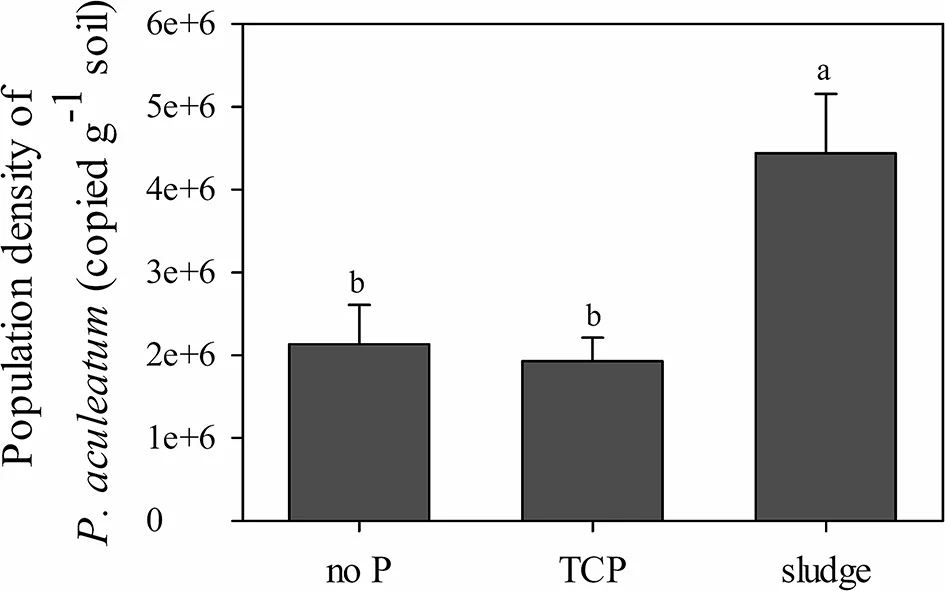
Population density of *P. aculeatum* found in soil at harvest (42 DAS) for plant inoculated with *P. aculeatum* (+ Pa) and amended with three different P sources (no P, tricalcium phosphate (TCP) and sewage sludge (sludge)). Values are the mean of four replicates, and error bars denote standard error. Different letters indicate significant treatment effects for each individual interaction

The abundance of *P. aculeatum* in soil at harvest for inoculated maize plant was in the range of 1.9 × 10^6^ – 4.4 × 10^6^ conidia g^− 1^ soil (Fig. 2), and thus significantly higher than the concentration of spores initially inoculated (2 × 10^5^ conidia g^− 1^ soil). The abundance of *P. aculeatum* in no P soil was not significantly different to the soil treated with Ca_3_(PO_4_)_2_, however, the addition of sewage sludge ash significantly increased the population density of *P. aculeatum* in soil.

The abundance of *P. aculeatum* was positively correlated with shoot height and plant biomass (Table 2), whereas there was a negative correlation between the abundance of *P. aculeatum* and *M. bolleyi* root infection.

### Plant nutrient content

Factor treatments mean of nutrient content quantified at harvest (42 DAS) in shoots and roots are presented in Table 3. No significant “Inoculum” × “P source” interactions were found for any of the shoot and root nutrients measured (Table S1 supplementary material). However, all macronutrients quantified (N, P, K, Ca, Mg and S) in shoot and the total amounts were significantly higher when maize plants were inoculated with *P. aculeatum* compared to maize plants without *P. aculeatum*. Similarly, the content of B, Cu, Mn and Zn in shoot and the total amount were also significant when maize plants were inoculated with *P. aculeatum*. However, no effect of inoculation was observed for any macro and micronutrients quantified in roots. The different P sources added also altered the amount of nutrients in shoot, root and total. Whereas no effect of addition of TCP was observed for any of the nutrients measured, the addition of sewage sludge ash increased all the macro and micronutrients measured in shoot, root and total compared to the no P treatment, except for shoot Fe content, which was not affected, and shoot Na content, which decreased with TCP addition. Shoot nutrient content and root Na were positively correlated with the abundance of *P. aculeatum*.

**Table 3:** Factor treatment means ± standard error of macro and micronutrients content measured in shoot, root, and total biomass at harvest (42 DAS) for plants non-inoculated (-Pa) or inoculated with *P. aculeatum* (+ Pa) and amended with three different P sources (no P, tricalcium phosphate (TCP) and sewage sludge (sludge)). Different letters indicate significant differences among treatments for each factor (-Pa vs. +Pa or No P vs. TCP vs. Sludge)

		*N*	*P*	K	Ca	Mg	S	B	Cu	Fe	Mn	Na	Zn
		mg						µg					
		Shoot											
Inoculum	-Pa	48 ± 4.8^b^	4.4 ± 1.0^b^	137 ± 15^b^	20 ± 1.8^b^	7.5 ± 1.1^b^	3.5 ± 0.3^b^	56 ± 4.8^b^	14 ± 1.9^b^	141 ± 14^a^	103 ± 8.6^b^	87 ± 21^a^	95 ± 13^b^
	+Pa	58 ± 4.5^a^	5.7 ± 1.2^a^	161 ± 13^a^	23 ± 1.5^a^	9.9 ± 1.2^a^	4.4 ± 0.3^a^	71 ± 5.7^a^	19 ± 2.1^a^	220 ± 48^a^	115 ± 7.5^a^	117 ± 23^a^	113 ± 12^a^
P source	No P	42 ± 2.8^b^	2.4 ± 0.2^b^	115 ± 8^b^	18 ± 1.7^b^	6.1 ± 0.6^b^	3.3 ± 0.3^b^	53 ± 3.2^b^	12 ± 1.1^b^	132 ± 15^a^	93 ± 5.1^b^	147 ± 37^a^	77 ± 5.1^b^
	TCP	43 ± 1.6^b^	2.7 ± 0.2^b^	119 ± 5^b^	18 ± 0.7^b^	6.0 ± 0.3^b^	3.2 ± 0.1^b^	52 ± 2.6^b^	12 ± 0.8^b^	152 ± 11^a^	90 ± 3.1^b^	114 ± 13^ab^	74 ± 3.1^b^
	Sludge	73 ± 3.3^a^	10.2 ± 0.6^a^	213 ± 4^a^	28 ± 1.3^a^	14.0 ± 0.7^a^	5.3 ± 0.3^a^	86 ± 5.2^a^	25 ± 1.6^a^	258 ± 71^a^	143 ± 5.9^a^	45 ± 9^b^	162 ± 3.8^a^
		Root											
Inoculum	-Pa	17.2 ± 2.7^a^	3.0 ± 1.0^a^	12.2 ± 2.6^a^	9.6 ± 2.0^a^	2.9 ± 0.6^a^	3.8 ± 0.8^a^	14.2 ± 2.4^a^	53 ± 13^a^	7602 ± 1545^a^	161 ± 26^a^	1191 ± 294^a^	103 ± 28^a^
	+Pa	19.5 ± 2.1^a^	2.6 ± 0.6^a^	13.5 ± 2.3^a^	9.8 ± 1.6^a^	3.1 ± 0.5^a^	4.1 ± 0.6^a^	14.2 ± 2.0^a^	57 ± 10^a^	7297 ± 1250^a^	150 ± 22^a^	1458 ± 291^a^	93 ± 17^a^
P source	No P	11.8 ± 1.2^b^	0.9 ± 0.1^b^	6.3 ± 0.8^b^	5.0 ± 0.5^b^	1.5 ± 0.2^b^	2.1 ± 0.2^b^	8.3 ± 1.1^b^	24 ± 2.7^b^	3762 ± 513^b^	89 ± 11^b^	584 ± 95^b^	40 ± 4.3^b^
	TCP	14.7 ± 1.4^b^	1.4 ± 0.2^b^	8.7 ± 1.2^b^	6.8 ± 0.7^b^	2.1 ± 0.2^b^	2.8 ± 0.3^b^	11.7 ± 1.2^b^	31 ± 3.7^b^	5656 ± 682^b^	131 ± 15^b^	763 ± 86^b^	57 ± 6.0^b^
	Sludge	28.6 ± 1.3^a^	6.1 ± 0.8^a^	23.5 ± 1.1^a^	17.3 ± 1.4^a^	5.4 ± 0.4^a^	7.1 ± 0.4^a^	22.7 ± 1.9^a^	108 ± 3.9^a^	12,930 ± 1377^a^	247 ± 23^a^	2626 ± 168^a^	198 ± 21^a^
		Total											
Inoculum	-Pa	65 ± 7.2^b^	7.4 ± 1.9^a^	150 ± 17^b^	29 ± 3.5^b^	10.4 ± 1.6^b^	7.3 ± 1.0^b^	70 ± 6.4^b^	67 ± 15^b^	7743 ± 1549^a^	264 ± 33^a^	1278 ± 285^b^	198 ± 40^a^
	+Pa	77 ± 6.3^a^	8.4 ± 1.8^a^	174 ± 15^a^	33 ± 2.9^a^	12.9 ± 1.7^a^	8.5 ± 0.9^a^	85 ± 7.5^a^	76 ± 12^a^	7517 ± 1262^a^	265 ± 28^a^	1575 ± 275^a^	206 ± 29^a^
P source	No P	54 ± 3.6^b^	3.3 ± 0.3^b^	122 ± 8.2^b^	23 ± 2.0^b^	7.5 ± 0.7^b^	5.4 ± 0.4^b^	61 ± 3.7^b^	36 ± 3.4^b^	3894 ± 515^b^	183 ± 12^b^	731 ± 100^b^	117 ± 8^b^
	TCP	57 ± 2.9^b^	4.1 ± 0.3^b^	128 ± 6.3^b^	25 ± 1.3^b^	8.0 ± 0.5^b^	6.0 ± 0.4^b^	63 ± 3.3^b^	44 ± 4.2^b^	5809 ± 678^b^	221 ± 16^b^	877 ± 93^b^	131 ± 8^b^
	Sludge	102 ± 2.6^a^	16.3 ± 0.6^a^	236 ± 5.0^a^	45 ± 1.1^a^	19.4 ± 0.7^a^	12.3 ± 0.3^a^	109 ± 4.2^a^	134 ± 2.9^a^	13,188 ± 1345^a^	391 ± 22^a^	2671 ± 162^a^	360 ± 19^a^

**Fig. 3:**
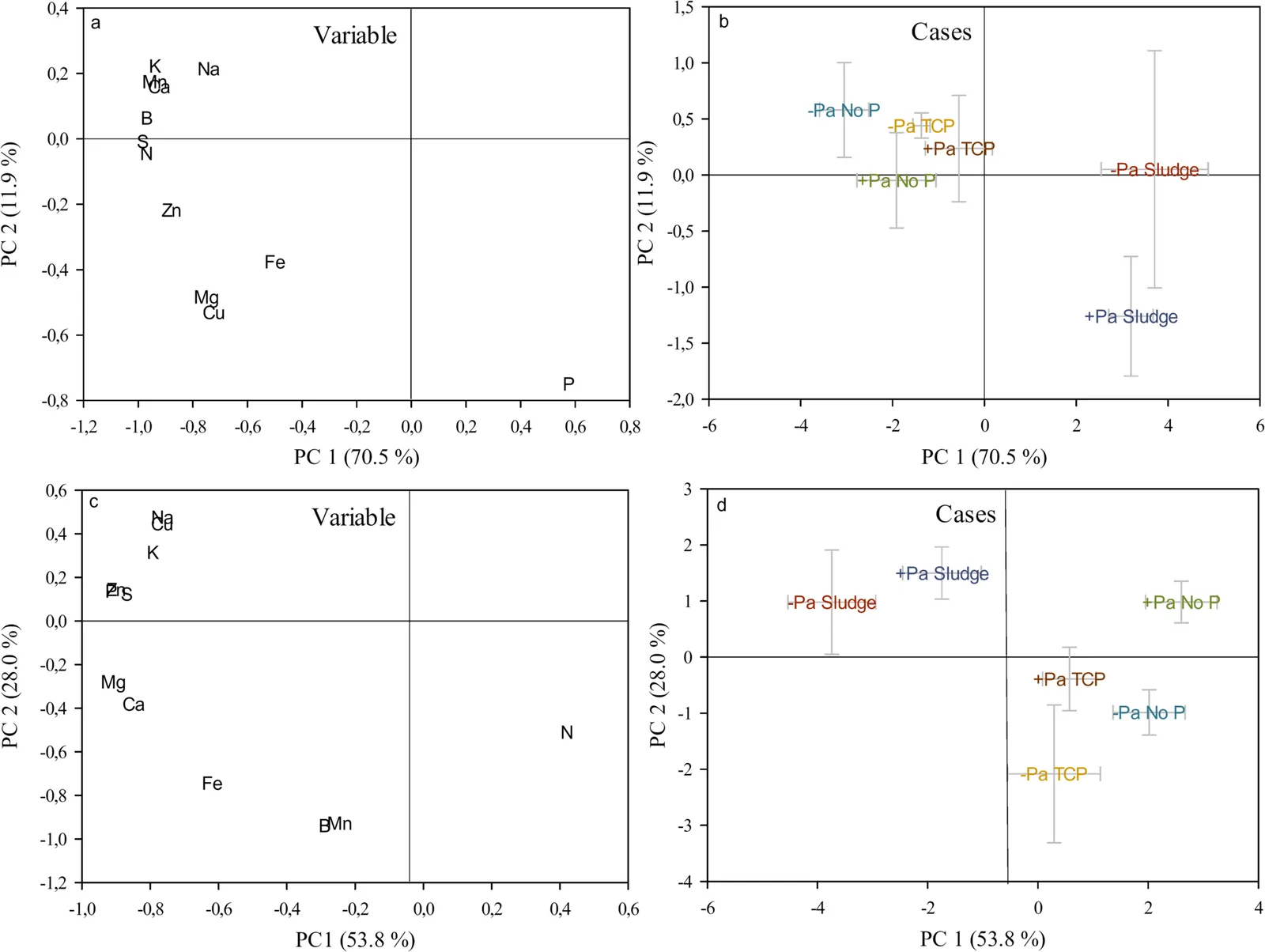
Principal component analysis results for the principal component 1 (PC 1) and 2 (PC 2) for shoot (a and b) and root (c and d) quantified at harvest (42 DAS) for plants inoculated with *P. aculeatum* (+ Pa) and amended with three different P sources (no P, tricalcium phosphate (TCP) and sewage sludge (sludge)). Values are the mean of four replicates, and error bars denote standard error

The principal component analyses of the nutrient composition performed for the shoot and root are shown in Fig. 3. For the shoots (Fig. 3a, b), the principal component 1 (PC1) explained 70.5% of the data variability, whereas PC2 explained 11.9%. PC1 was significantly positively correlated with P and negatively with the rest of macro and micronutrients. The scatter plot showed a clear separation of the experimental units for PC1 and PC2 according to the P source added. Shoot experimental units fertilized with sewage sludge ash were in the positive area of PC1, followed by Ca_3_(PO_4_)_2_ and no P treatments, which were both located in the negative area of PC1. For the roots (Fig.3c, d), 53.8% of the data variability was found for PC1 and 28% for PC2. PC1 was positively correlated with N and negatively with the rest of macro and micronutrients. In this case, the distribution of the experimental units was also driven by the source of P added, which were clearly separated according to PC1, being no P treatments located in the positive area of PC1, followed by Ca_3_(PO_4_)_2_ and sewage sludge ash that was in the negative area of PC1.

## Discussion

Here we show that inoculation with *P. aculeatum*, pre-activated with cellulose as a C source to improve its establishment and persistence in natural agricultural soil, resulted in improved maize plant growth and nutrition independent of the type of P source tested. Also, inoculation with *P. aculeatum* resulted in increased root colonization with native populations of AMF and reduction of infection with the root-inhabiting fungus *M. bolleyi*. These results confirm the hypothesis that application of *P. aculeatum* pre-activated with cellulose would lead to the promotion of maize plant growth and P nutrition, and alteration in the AMF and *M. bolleyi* root association.

However, surprisingly the maize plant growth promotion with *P. aculeatum* was independent (there was not a significant interaction between “Inoculation” and “P source”) of the P fertilization scenario studied (without P fertilization, Ca_3_(PO_4_)_2_ or sewage sludge ash), although P concentration in shoot was significantly higher in plant treated with sewage sludge ash compared to the other two treatments. These results suggest that a mechanism of action other than P solubilization may be involved in the observed plant growth and nutrient promotion by *P. aculeatum*.

Our results showing a strong negative correlation between maize plant growth variables measured and *M. bolleyi* root infection, suggesting that in the present study *M. bolleyi* could be related with maize growth depression. *M. bolleyi* is a common root-inhabiting fungus in cereals [31] such as wheat [32, 33], barley [34] and maize [23, 35]. In terms of functional traits, *M. bolleyi* has been reported as a pathogen in barley [34], but also as a biocontrol agent against the take-all disease in wheat caused by *Gaumannomyces graminis* [36]. This highlights its context-dependent ecological roles.

Interestingly, in the present study inoculation with *P. aculeatum* coincided with a clear reduction in *M. bolleyi* root infection. This decrease may have contributed to alleviating potential negative effects associated with *M. bolleyi*, resulting in improved maize growth. This pattern suggests that *P. aculeatum* could have acted as a biocontrol agent against *M. bolleyi* under our experimental conditions. Indeed, different species of *Penicillium* are known biocontrol agents of root diseases caused by *Fusarium* spp. and other root pathogens [37, 38], but to our knowledge, this is the first report indicating a potential biocontrol of *M. bolleyi* by *Penicillium* spp. However, the underlying mechanisms for this biocontrol trait of *P. aculeatum* should be further investigated.

Along the same line, the observed increase in AMF root colonization after *P. aculeatum* inoculation could be another explanation for the maize plant growth and nutrition promotion by *P. aculeatum*, though in this case no correlations between AMF root colonization and plant growth and nutrition were found. Recently, Elgharably et al. [39] reported how the combined inoculation with *Penicillium* and AMF was able to increase wheat growth and nutrient uptake. Maize naturally associates with native communities of AMF, which often promote host growth and nutrition [40, 41], though this promotion depends on soil P level and type of fertilizer [42] and other agricultural practices [43]. However, in the present study, the different P fertilizers examined had no effects on AMF root colonization, most likely because both P sources are not readily available as phosphate, which is the main factor determining AMF root colonization in maize [40].

The observed increase in almost all measured plant nutrients in terms of total content with *P. aculeatum* inoculation is logical since it coincides with an overall increase in maize biomass production. However, it is interesting that this was the case for total shoot nutrient content, but not for root nutrient content. Our results showing that both P fertilization and *P. aculeatum* altered the composition of nutrient composition in maize shoot and root calls for more studies on the role of *Penicillium* spp. as biofertilizers not only in terms of plant phosphorus acquisition but also other macro and micronutrients [44]. This is also important to consider when evaluating the use of sewage sludge ash as a general macro and micronutrient fertilizer.

Our results showing increased population density of *P. aculeatum* in combination with sewage sludge ash are in accordance with other studies on different types of biochar including sewage sludge ash [45]. The underlying mechanism for this cannot be revealed in this study but could be related to improved conditions as a carrier and refugium for fungal survival and growth [46].

Our results showing reduced *M. bolleyi* root infection with both examined P fertilizers coincide with improved plant growth. However, information about the effects of fertilizers on root infection with *M. bolleyi* is limited [23], but this should be further addressed to unravel the mechanisms involved.

In conclusion, here we demonstrated that inoculation with *P. aculeatum* pre-activated with cellulose and P sources prior to inoculation to natural non-sterile agricultural soil can promote plant growth and health in maize. Surprisingly, the effect of *P. aculeatum* was independent of the P sources evaluated. Therefore, we propose an alternative mode of action of plant growth promotion by *P. aculeatum* other than P solubilisation, based on possible biocontrol traits against the root pathogen *M. bolleyi.* This should be further investigated with studies on interactions between *P. aculeatun* and *M. bolleyi* under controlled conditions.

## Supplementary Information

Below is the link to the electronic supplementary material.

Supplementary Material 1

## Data Availability

No datasets were generated or analysed during the current study.
