# Supplementary material for: Penicillium aculeatum pre-activated with cellulose and different phosphorus sources promotes maize growth, nutrition and mycorrhiza formation, and decreases Microdochium bolleyi root infection
Source: Curr Microbiol. 2026 Aug 26;83(10):537. doi: 10.1007/s00284-026-05098-4 (PMC13518440; doi:10.1007/s00284-026-05098-4)
Supplement: Supplementary file 1 [file 284_2026_5098_MOESM1_ESM.docx]

Table S1. Results of factorial ANOVA *p-values* from single factors and their interactions for all variables measured in this study

|  |  | **Factors** | | | | | |  |
| --- | --- | --- | --- | --- | --- | --- | --- | --- |
| **Variables** | | | **Inoculo** | | **Psource** | | **Inoculo**×**Psource** | |
| **Shoot**  **height** | 14 | 0.669 | | 0.292 | | 0.659 | |  |
|  | 22 | 0.091 | | 0.038 | | 0.123 | |  |
|  | 29 | 0.007 | | 0.000 | | 0.179 | |  |
|  | 42 | 0.000 | | 0.000 | | 0.057 | |  |
| **Root**  **length** | Total | 0.164 | | 0.000 | | 0.233 | |  |
|  | Specific | 0.330 | | 0.254 | | 0.338 | |  |
| **Biomass** | Shoot | 0.002 | | 0.000 | | 0.797 | |  |
|  | Root | 0.064 | | 0.000 | | 0.525 | |  |
|  | Total | 0.004 | | 0.000 | | 0.849 | |  |
|  | Root:shoot | 0.206 | | 0.409 | | 0.155 | |  |
| **Fungi** | AMF | 0.000 | | 0.222 | | 0.939 | |  |
|  | *M. bolleyi* | 0.000 | | 0.000 | | 0.034 | |  |
|  | *P. aculeatum*  abundance | 0.000 | | 0.005 | | 0.005 | |  |
| **Shoot** | N | 0.000 | | 0.000 | | 0.642 | |  |
|  | P | 0.000 | | 0.000 | | 0.101 | |  |
|  | K | 0.000 | | 0.000 | | 0.416 | |  |
|  | Ca | 0.029 | | 0.000 | | 0.352 | |  |
|  | Mg | 0.000 | | 0.000 | | 0.096 | |  |
|  | S | 0.000 | | 0.000 | | 0.540 | |  |
|  | B | 0.000 | | 0.000 | | 0.440 | |  |
|  | Cu | 0.000 | | 0.000 | | 0.597 | |  |
|  | Fe | 0.110 | | 0.089 | | 0.418 | |  |
|  | Mn | 0.036 | | 0.000 | | 0.784 | |  |
|  | Na | 0.279 | | 0.021 | | 0.675 | |  |
|  | Zn | 0.000 | | 0.000 | | 0.564 | |  |
| **Root** | N | 0.113 | | 0.000 | | 0.148 | |  |
|  | P | 0.539 | | 0.000 | | 0.279 | |  |
|  | K | 0.277 | | 0.000 | | 0.297 | |  |
|  | Ca | 0.850 | | 0.000 | | 0.428 | |  |
|  | Mg | 0.656 | | 0.000 | | 0.652 | |  |
|  | S | 0.462 | | 0.000 | | 0.160 | |  |
|  | B | 0.984 | | 0.000 | | 0.773 | |  |
|  | Cu | 0.251 | | 0.000 | | 0.035 | |  |
|  | Fe | 0.795 | | 0.000 | | 0.869 | |  |
|  | Mn | 0.600 | | 0.000 | | 0.824 | |  |
|  | Na | 0.069 | | 0.000 | | 0.981 | |  |
|  | Zn | 0.476 | | 0.000 | | 0.053 | |  |
| **Total** | C | 0.002 | | 0.000 | | 0.792 | |  |
|  | S | 0.002 | | 0.000 | | 0.238 | |  |
|  | Mg | 0.000 | | 0.000 | | 0.676 | |  |
|  | Cu | 0.011 | | 0.000 | | 0.042 | |  |
|  | Mn | 0.967 | | 0.000 | | 0.769 | |  |
|  | Zn | 0.552 | | 0.000 | | 0.058 | |  |
|  | Ca | 0.043 | | 0.000 | | 0.306 | |  |
|  | Fe | 0.845 | | 0.000 | | 0.888 | |  |
|  | K | 0.000 | | 0.000 | | 0.426 | |  |
|  | Na | 0.041 | | 0.000 | | 0.954 | |  |
|  | P | 0.052 | | 0.000 | | 0.851 | |  |
|  | N | 0.000 | | 0.000 | | 0.480 | |  |
